# Novel oscillator model with damping factor for plasmon induced transparency in waveguide systems

**DOI:** 10.1038/s41598-017-11335-5

**Published:** 2017-09-06

**Authors:** Mingzhuo Zhao, Hongjian Li, Zhihui He, Zhiquan Chen, Hui Xu, Mingfei Zheng

**Affiliations:** 1 School of Physics and Electronic, Central South University, Changsha, 410083 PR China; 2 School of Physics and Electronic, Hunan University of Science and Technology, Xiangtan, 411201 PR China

## Abstract

We introduce a novel two-oscillator model with damping factor to describe the plasmon induced transparency (PIT) in a bright-dark model plasmonic waveguide system. The damping factor γ in the model can be calculated from metal conductor damping factor γ_*c*_ and dielectric damping factor γ_*d*_. We investigate the influence of geometry parameters and damping factor γ on transmission spectra as well as slow-light effects in the plasmonic waveguide system. We can find an obvious PIT phenomenon and realize a considerable slow-light effect in the double-cavities system. This work may provide guidance for optical switching and plasmon-based information processing.

## Introduction

Electromagnetically induced transparency (EIT) is an appealing physical phenomenon where the otherwise opaque medium becomes transparent for a probe laser modified by a coupling laser beam^1^, which is the elimination of absorption over a narrow spectral region in a broad absorption regime and accompanied with steep dispersion^2, 3^. This strong dispersion plays a key role to reduce the group velocity of light^4–7^. But it requires complicated experimental handling because of the rather short coherence times of the superposition state. It is soon found that the characteristic features such as low absorption and steep dispersion can also be realized in classical systems such as plasmonic structures^8–17^. Recently, Zeng *et al*. reported tunable multiple phase-coupled plasmon induced transparencies in grapheme metamaterials^18^. Zeng *et al*. reported high-contrast electro-optic modulation of spatial light induced by graphene-integrated Fabry-Pérot microcavity^19^. Xu *et al*. studied the PIT transmission by a metal-insulator-metal (MIM) bus waveguide coupled with a single defective cavity based on the coupled mode theory (CMT)^20^. He *et al*. reported the aspect ratio control and sensing applications for metal–dielectric–metal (MDM) slot waveguides with a multimode stub through a radiation field model (RFM)^21^. He *et al*. first introduced the two-oscillator model to describe the PIT in bright-dark mode plasmonic waveguide systems^22^. However, the damping factor in both CMT and RFM are always first obtained from the simulation data before seeking transmission and scattering parameters.

In this paper, we approximately calculate the damping factor γ by introducing the metal conductor damping γ_*c*_ and dielectric damping γ_*d*_. Transmission characteristics, slow-light effects based on coupling strength κ, geometry parameters, and damping factor γ are discussed in detail. We find that the theoretical results are in agreement with the Finite-Difference Time-Domain (FDTD) simulations. This research may provide a new way to study PIT in the plasmonic waveguide systems and it also can give a theoretical guidance for plasmon-based information processing.

## Plasmonic systems and theoretical model

**Figure 1:**
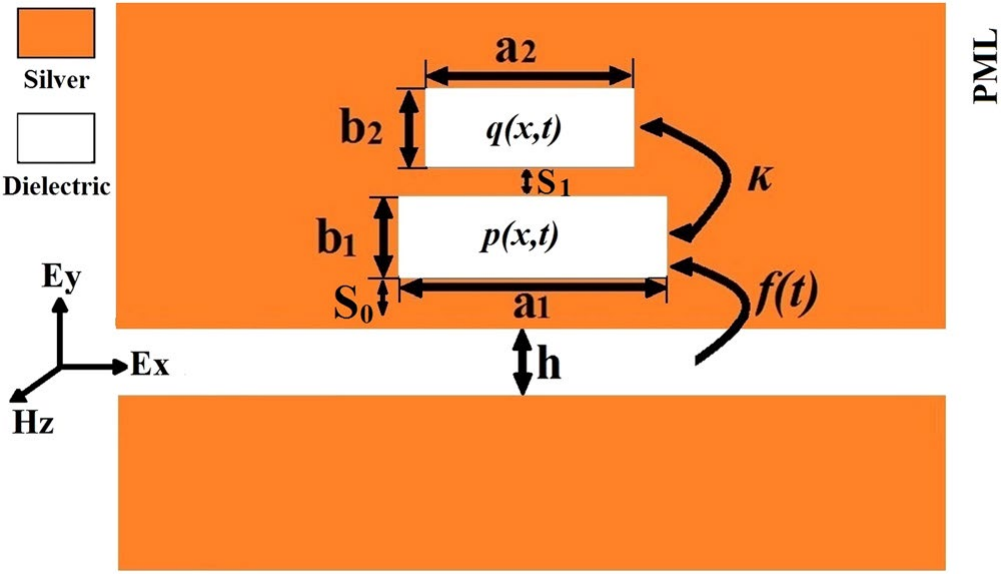
Schematic of a MDM waveguide side-coupled with two rectangular resonators with h = 80 *nm* and s_0_ = s_1_ = 20 *nm*.

We consider a metal-dielectric-metal (MDM) waveguide coupling with rectangular resonators with h = 80 *nm*, s_0_ = s_1_ = 20 *nm* as shown in Fig. 1. The metal is silver (Ag). The frequency dependent optical property of the silver nanostructure is approximated by the Drude model ε(ω) = ε_∞_−ω_p_^2^/(ω^2^ + iωγ_p_)^23^, ω_p_ = 1.38 × 10^16^ rad/s is the bulk plasmon frequency, ε_∞_ = 3.7 and γ_p_ = 2.37 × 10^13^rad/s represents the damping rate. The calculated area is divided into uniform Yee cells and surrounded by perfectly matched layer (PML) absorbing boundary. The bottom resonator is considered as the bright mode, which is directly excited by the input pulse. The top resonators which can’t be directly excited by the input pulse rather than driven by the bottom resonator are considered as dark modes^14^.

Here, we regard the two rectangular resonators as a set of two coupled harmonic oscillators^22^.

1 $${{\omega }_{1}}^{-2}\ddot{p}(x,t)+{\gamma }_{1}{{\omega }_{1}}^{-1}\dot{p}(x,t)+p(x,t)=f(x,t)-\kappa q(x,t)$$

2 $${{\omega }_{2}}^{-2}\ddot{q}(x,t)+{\gamma }_{2}{{\omega }_{2}}^{-1}\dot{q}(x,t)+q(x,t)=-\kappa p(x,t)$$

where damping factor γ_1_ and γ_2_ are described by the excitation *p(x,t)* and *q(x,t)*, respectively. *p(x,t)* is driven by *f(x,t)*. The two resonators are linearly coupled with coupling strength *κ*.

In order to investigate the rectangular resonator specifically, we introduce a method to calculate the damping factor γ, which is mainly constrained by metal conductor damping γ_*c*_ and dielectric damping γ_*d*_, the total damping factor is γ = γ_*d*_ + γ_*c*_. Damping caused by metal conductor loss depends on the field distribution in the resonator, so it must be evaluated separately for each type of resonators. It is well known that each metal-dielectric interfaces supports a localized surface plasmon polaritons (SPPs) mode propagating along the x. If the distance between the interface is comparable to or smaller than the skin depth of SPPs in dielectric, the localized modes become coupled. The coupled SPPs modes of frequency ω are described by the electromagnetic field components *U(x,y,t)* = *[E*_*x*_ *,E*_*y*_ *,H*_*z*_]. For obtaining the intrinsic metal damping γ_*c*_, it is better to use the perturbation method. The power lost per unit length due to finite wall conductivity is^24^

3 $${P}_{l}=\frac{{R}_{S}}{2}{{\oint }_{C}|{H}_{z}|}^{2}dl$$

where R_s_ is the wall surface resistance, and the integration contour C encloses the inside perimeter of the resonator walls. There are surface currents on all walls.

It is instructive to compute the Poynting vector to see how power propagates in the TM_m_ mode. According to the electromagnetic theory, the time-average power storing in a rectangular cavity is

4 $$P=\frac{Z}{2}{{\iint }_{s}|{H}_{z}|}^{2}dS$$

where Z is the wave impedance. According to electromagnetic theory and Eqs (3–4), the metal damping factor γ_c_ in the rectangular resonators can be written in following form

5 $${\gamma }_{c}=\frac{{P}_{l}}{2P}=\frac{{R}_{s}}{Z}(\frac{2}{b}+\frac{1}{a})$$

However, if the rectangular resonator is completely filled with a homogeneous dielectric, the damping for a lossy dielectric material can be calculated from the propagation constant, and this result will apply to any resonator with a homogeneous dielectric filling. Thus, the complex permittivity allows the complex propagation constant to be written as

6 $$\begin{array}{rcl}\beta  & = & {\gamma }_{d}+i\alpha \\  & = & \sqrt{{{k}_{d}}^{2}-{k}^{2}}\\  & = & \sqrt{{{k}_{d}}^{2}-{\omega }^{2}{\mu }_{0}{\varepsilon }_{0}{\varepsilon }_{d}(1-j\,\tan \,\delta )}\end{array}$$

In practice, most dielectric materials have small losses (loss tangent tan δ << 1), and this expression can be simplified by using the first two terms of the Taylor expansion

7 $$\sqrt{{d}^{2}+{x}^{2}}\approx d+\frac{1}{2}(\frac{{x}^{2}}{d}),\quad \quad for\,\,x\ll d$$

Then Eq. (6) reduces to

8 $$\begin{array}{rcl}\beta  & = & \sqrt{{{k}_{d}}^{2}-{k}^{2}+j{k}^{2}\,\tan \,\delta }\\  & \approx  & \sqrt{{{k}_{d}}^{2}-{k}^{2}}+\frac{j{k}^{2}\,\tan \,\delta }{2\sqrt{{{k}_{d}}^{2}-{k}^{2}}}\\  & = & \frac{{k}^{2}\,\tan \,\delta }{2\alpha }+j\alpha \end{array}$$

where $\sqrt{{{k}_{d}}^{2}-{k}^{2}}=j\alpha$. In these results, $k=\omega \sqrt{{\mu }_{0}{\varepsilon }_{0}{\varepsilon }_{1}}$ is the real wave number in the absence of loss. Eq. (8) shows that the phase constant α is unchanged when the loss is small while the damping constant due to dielectric loss is given by

9 $${\gamma }_{d}=\frac{{k}^{2}\,\tan \,\delta }{2\alpha }$$

The total damping factor can be shown in the following form

10 $$\gamma =\frac{{R}_{s}}{Z}(\frac{2}{b}+\frac{1}{a})+\frac{{k}^{2}\,\tan \,\delta }{2\alpha }$$

In order to model scattering parameters of PIT response, electric current sheet with surface conductivity *σ*_*se*_ may describe this effective response. The scattering parameters of an electric current sheet are^24^

11 $$T=\frac{2}{2+\zeta {\sigma }_{se}}$$

where ζ is the wave impedance of the external waves. We can now determine the surface conductivity from the constitutive equation

12 $${\sigma }_{se}\approx \frac{-i\omega [1-{(\omega /{\omega }_{2})}^{2}-i{\gamma }_{2}(\omega /{\omega }_{2})]}{[1-{(\omega /{\omega }_{1})}^{2}-i{\gamma }_{1}(\omega /{\omega }_{1})][1-{(\omega /{\omega }_{2})}^{2}-i{\gamma }_{2}(\omega /{\omega }_{2})]-{\kappa }^{2}}$$

Once we have determined the surface conductivity, we can calculate the scattering parameters from Eq. (11) and other derived quantities, such as transmission phase and group index

13 $$\phi (\omega )=\text{arg}(T)$$

14 $${n}_{g}=-\frac{1}{2}\frac{c}{L}\text{Im}(T\frac{d\zeta {\sigma }_{se}}{d\omega })$$

where c is the velocity of the light, and L = 700 *nm* is the length of the bus waveguide.

**Figure 2:**
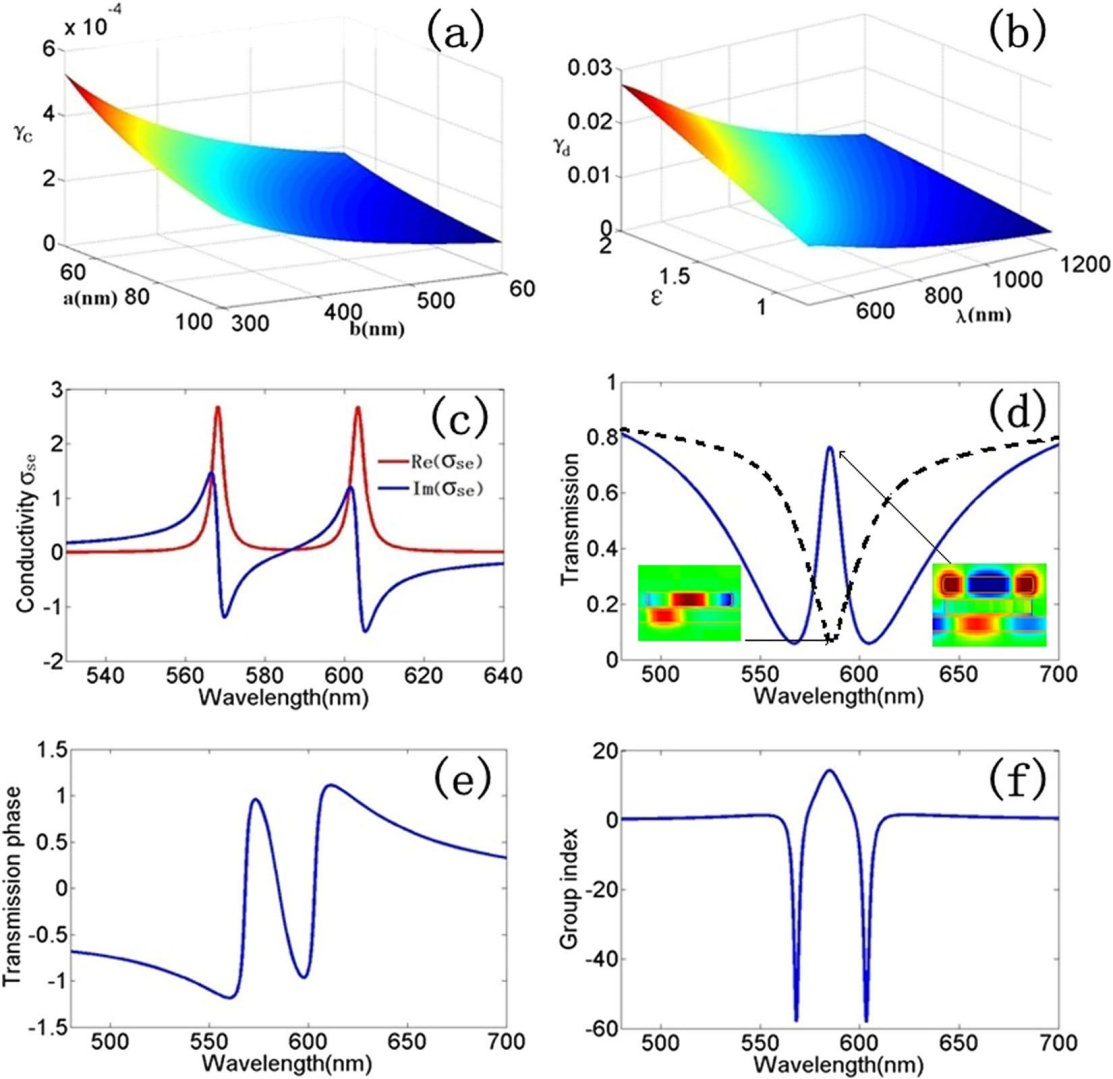
(**a**) The relationship between γ_c_ and geometry parameters of resonators. (**b**) The trends of γ_d_ due to the wavelength λ and permittivity ε. (**c**–**f**) represent the spectra of surface conductivity, transmission, phase and group index of plasmonic analog of EIT as a function of wavelength in plasmonic waveguide system, respectively. Where it is described by the radiating two-oscillator mode with γ_1_ = γ_2_ = 0.006 and κ = 0.06.

The radiating two-oscillator model allows us to understand the response of PIT in plasmonic waveguide systems. In Fig. 2, we plot the γ_d_, γ_c_, the surface conductivity, the transmission amplitude and phase and the group index n_g_ for a set of parameters. Figure 2(a) shows the relationship between γ_c_ and geometry parameters of resonators, we can see that the γ_c_ changes tardily with the increasing of the length and width of resonators, moreover, the value level of γ_c_ is about 10^−4^. The trends of γ_d_ as a function of the wavelength λ and permittivity ε are shown in Fig. 2(b). In Fig. 2(c–f), we recognize the typical features of PIT. The conductivity has enveloped with sharp incisions, resulting in a frequency window with large transmission at the resonance frequency. At the same time, there is large normal dispersion in the transmission phase, which leads to a significantly enhanced group delay. The reduced response can be understood from the destructive interference of the excitation due to the external field and the coupling with the dark resonator.

## Transmission characteristics and slow-light effects

**Figure 3:**
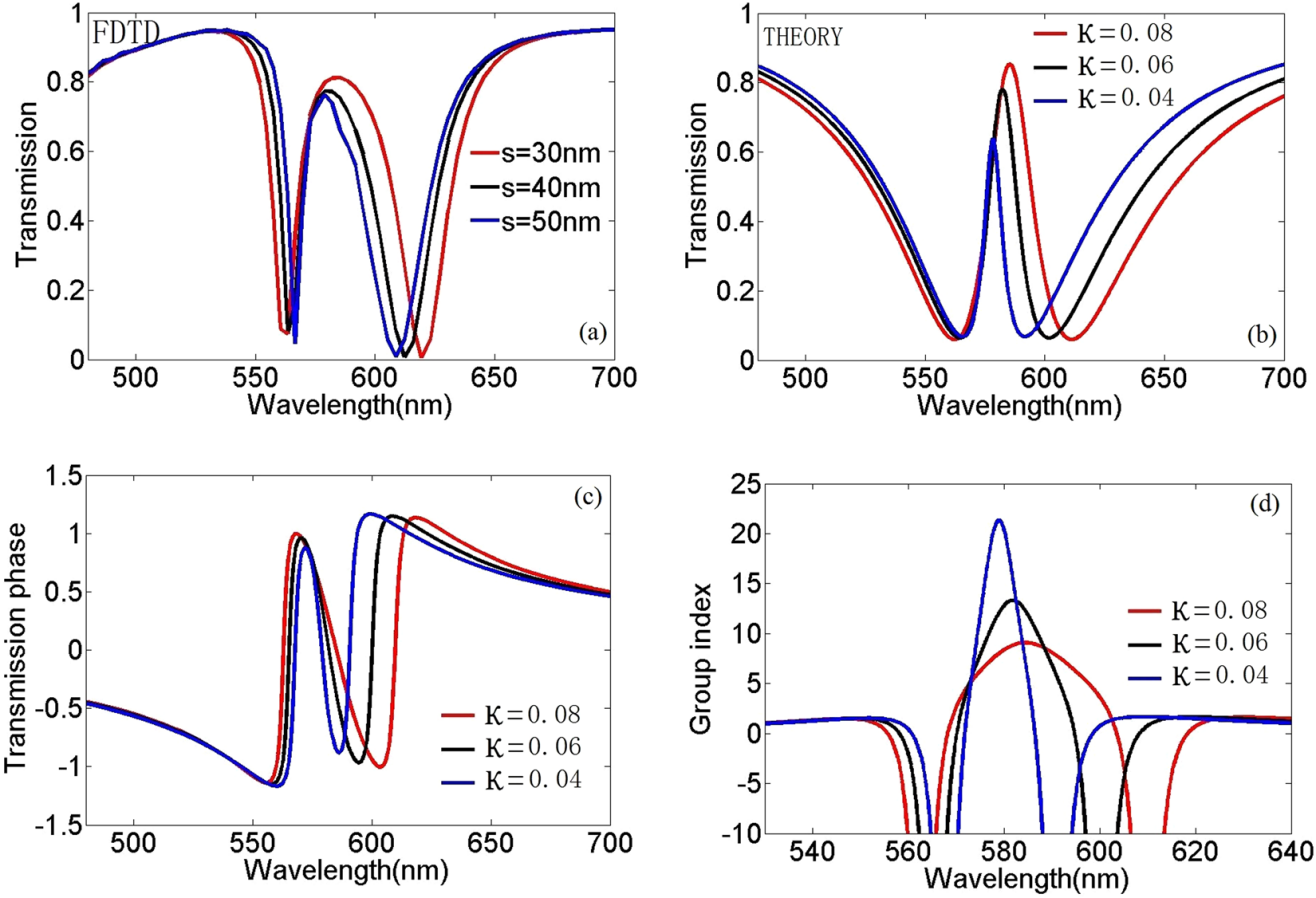
(**a**) Transmission spectra of FDTD simulations in plasmonic waveguide systems. (**b**) Transmission spectra of theory data in plasmonic waveguide systems. (**c**–**d**) Transmission phase and group index of the plasmonic analog of EIT in plasmonic waveguide system for different coupling strengths with γ_1_ = γ_2_ = 0.006, respectively.

Coupling strength κ is a critical factor for PIT phenomenon. It is determined by effective coupling distance, material parameters and so on. We investigate the transmission characteristics as a function of coupling strength κ with κ^2^»γ_1_γ_2_^25^ in both theory and simulation as shown in Fig. 3(a) and (b), we can see that the theoretical transmission simulation [Fig. 3(a)] is in agreement with the FDTD simulation [Fig. 3(b)] in transmission spectrum. Figure 3(c) and (d) show the transmission phase and group index in different coupling strength κ. We find that a weak coupling strength κ will have a big group index, so we can adjust the distance between resonant cavities to control the speed of light.

**Figure 4:**
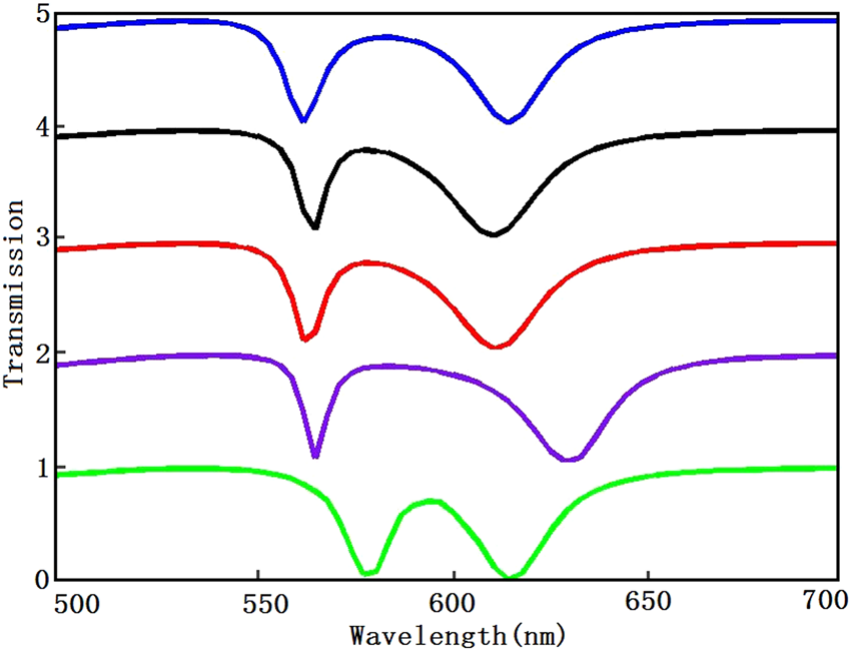
Transmission spectra in plasmonic waveguide systems for different geometry parameters. Blue line with parameters a_1_ = a_2_ = 400 nm, b_1_ = 100 nm and b_2_ = 80 nm, black line with parameters a_1_ = a_2_ = 400 nm, b_1_ = 80 nm and b_2_ = 100 nm, red line with parameters a_1_ = a_2_ = 400 nm, b_1_ = b_2_ = 80 nm, purple line with parameters b_1_ = b_2_ = 80 nm, a_1_ = 420 nm and a_2_ = 400 nm and green line with parameters b_1_ = b_2_ = 80 nm, a_1_ = 400 nm and a_2_ = 420 nm.

Based on the above studies, we plot the transmission spectrum of rectangular resonators in different geometry parameters of resonators as shown in Fig. 4. The blue line shows the transmission spectrum of plasmonic waveguide systems with parameters a_1_ = a_2_ = 400 nm, b_1_ = 100 nm and b_2_ = 80 nm. The black line with a_1_ = a_2_ = 400 nm, b_1_ = 80 nm and b_2_ = 100 nm. The red line with a_1_ = a_2_ = 400 nm, b_1_ = 80 nm and b_2_ = 80 nm. The purple line with b_1_ = b_2_ = 80 nm, a_1_ = 420 nm and a_2_ = 400 nm, and the green line with b_1_ = b_2_ = 80 nm, a_1_ = 400 nm and a_2_ = 420 nm. By comparing the red, blue and black line, we find that the transmission spectra are almost unchanged. However, when the two rectangular resonators have the same width and the different length, the second resonance dip is red shifted by increasing the length of the bright mode resonator, and the first resonance dip incur red shift by increasing the length of the dark mode resonator after making a comparison between the red, purple and green lines. Based on the Eq. (5), the damping factor γ_c_ decreases with the increasing of the width and length of rectangular resonator, but the value of the damping factor γ_c_ is too small and the geometry parameters of resonators have little effect on damping factor γ_c_, so the resonance wavelength of the resonators is determined by the length of the resonators.

**Figure 5:**
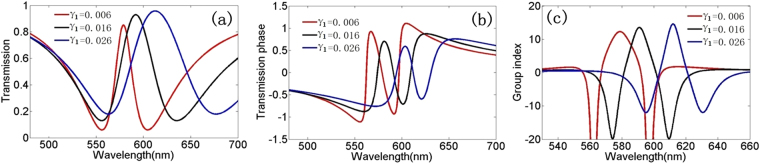
(**a**) Transmission spectra of theoretical data in plasmonic waveguide systems.(**b**–**c**). Transmission phase and group index in plasmonic waveguide systems for different γ_1_ in superradiant mode resonator with κ = 0.06 and γ_2_ = 0.006.

Then, we investigate the transmission characteristics and slow-light effects as the damping factor γ_1_ in bright mode resonator increases. In Fig. 5(a), we plot the theoretical transmission spectrum as a function of the damping factor γ_1_ of the bright mode resonator. We can find that the transmission window becomes broad, and the peak of the transmission spectrum increases with the increasing of γ_1_. Figure 5(b) and (c) show the transmission phase and group index with the increasing of the damping factor γ_1_, we can find that a large damping factor γ_1_ will have a large group index.

**Figure 6:**
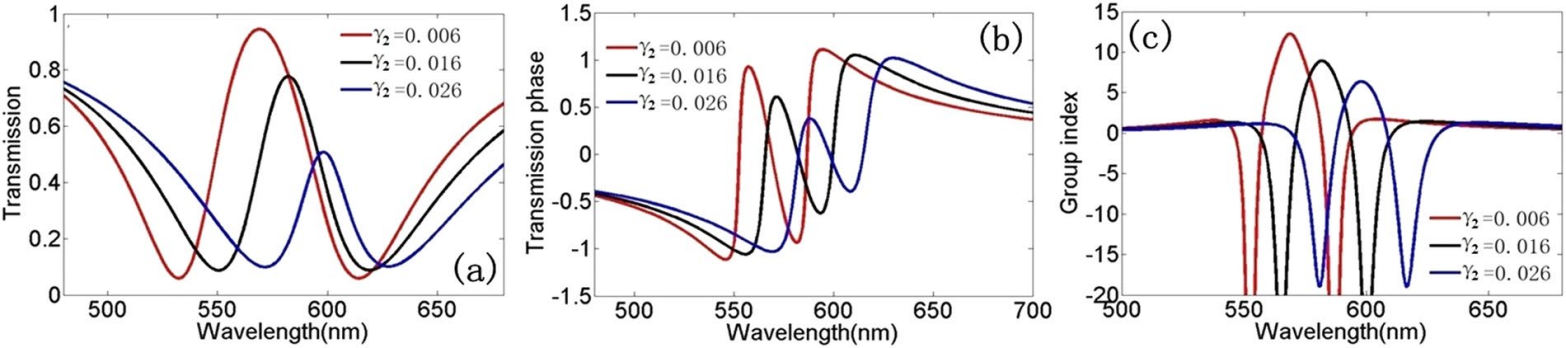
(**a**) Transmission spectrum of theoretical data in plasmonic waveguide systems. (**b**)–(**c**) Transmission phase and group index in plasmonic waveguide system for different γ_2_ in subradiant mode resonator with κ = 0.06 and γ_1_ = 0.006.

At last we research the transmission characteristics and slow-light effects of this system when the damping factor γ_2_ in dark mode resonator increases. Figure 6(a) shows the theoretical simulation transmission spectrum as a function of the damping factor γ2. With the damping factor increasing the transmission window of the PIT gets narrower and the transmission peak gets lower. Figure 6(b) and (c) show the transmission phase and group index with the increasing of the damping factor γ_2_, and we can find that a large damping factor γ_2_ will have a small group index. We can find an interesting phenomenon that the dark mode resonator lead to a counter transmission characteristics when the damping factor γ_2_ increases in bright mode resonator. This phenomenon can be explained by Eqs (11–14). We can adjust the damping factor γ of the resonant cavities to control the speed of light so that it facilitates study of photonic devices.

## Conclusion

To summarize, the PIT spectral response can be realized in the bright-dark model plasmonic waveguide system. The physical mechanism of it is elucidated well by a two-oscillator model through the introduction of rigorous damping factor γ and coupling coefficient κ. In order to investigate geometry parameters of the resonators and damping factor γ effect on transmission characteristics and scattering parameters, we discuss the damping factor γ through leading into the intrinsic metal damping γ_*c*_ and dielectric damping γ_*d*_ in detail. Our research may provide a new way to study the PIT in the plasmonic waveguide resonators.

## Methods

The frequency dependent optical property of the silver nanostructure is approximated by the Drude model ε(ω) = ε_∞_−ω_p_^2^/(ω^2^ + iωγ_p_), with ω_p_ = 1.38 × 10^16^ rad/s is the bulk plasmon frequency, ε_∞_ = 3.7 and γ_p_ = 2.37 × 10^13^rad/s represents the damping rate. The characteristic spectra of the structures are found by using the two-dimensional FDTD method with mesh grid size Δx = Δy = 5 nm. The Gauss light source is set at the entrance of the bus waveguide, and a normalized receiving screen is placed at the exit of the bus waveguide. The calculated domain is surrounded by perfectly matched layer absorbing boundary. We choose Meep as our FDTD simulation software developed by MIT. And the simulation parameters have been given in our paper.
